# Pelvic peritoneum reconstruction using the bladder peritoneum flap in laparoscopic extralevator abdominoperineal excision: A multi-center, prospective single-arm cohort study (IDEAL Phase 2A)

**DOI:** 10.1097/MD.0000000000020712

**Published:** 2020-06-19

**Authors:** Yu Shen, Tinghan Yang, Xiangbing Deng, Jinliang Yang, Wenjian Meng, Ziqiang Wang

**Affiliations:** a Department of Gastrointestinal Surgery; b State Key Lab of Biotherapy and Cancer Center, West China Hospital, Sichuan University, Chengdu, Sichuan, China.

**Keywords:** APR, bladder peritoneum flap, extralevator abdominoperineal excision, pelvic peritoneum reconstruction, rectal cancer

## Abstract

**Introduction:**

Extralevator abdominoperineal excision (ELAPE) may cause various surgical complications including disruption of perineal wound, perineal hernia and adhesive small-bowel obstruction. Pelvic peritoneum reconstruction (PPR) could prevent those complications, but it may not always be achievable, especially in patients with severe pelvic fibrosis after neoadjuvant radiotherapy. Our previous study has reported the application of the PPR using the bladder peritoneum flap in laparoscopic ELAPE. The aim of the study is to evaluate the short-term clinical, technical and safety outcomes of PPR using the bladder peritoneum flap in laparoscopic ELAPE.

**Methods and analysis:**

This is a multi-center prospective single-arm cohort study and fulfill the IDEAL 2A stage principle. Rectal cancer patients undergoing laparoscopic ELAPE, suffering rigid pelvis or huge perineal peritoneum defect, and having difficulty in primary perineal wound closure will be considered eligible. Main exclusion criteria are being complicated with urgent complications, ASA grade >3 and accompanied with mental illness. After informed consent, 30 patients are planned to be included in the study. Standard laparoscopic ELAPE with pelvic peritoneal floor reconstruction using bladder peritoneum flap are to be performed. The surgical safety is to be evaluated after one-year follow-up. Primary endpoints are the occurrence of intraoperative and postoperative complications of PPR using the bladder peritoneum flap. Second endpoints are overall complication rate within 30 days after surgery, extent of small intestine falling down to pelvic cavity, and other follow-up consequences within 1 year after surgery.

**Ethics and dissemination:**

This experiment was approved by the Biomedical Ethics Committee of West China Hospital of Sichuan University.

**Trial registration:**

NCT04177407.

## 1 Introduction

In rectal cancers, the overall survival will benefit strongly if a negative circumferential margin is reached. In order to pursue a negative margin, the extralevator abdominoperineal excision (ELAPE), which was introduced by Holm et al^[1]^ have been used to improve the oncological outcome in T3 and T4 low rectal cancer.^[2]^ However, after removal of the rectum and mesorectum, ELAPE leaves a dead pelvic space, which may allow the intraabdominal contents to descend. When the small bowel protrudes into the dead space and adhere to the pelvis, the risk for intestinal obstruction rises.^[3]^ In addition, the descending small bowel may further herniate through the perineal wound, leading to perineal hernia (PH). The downward pressure of intraabdominal content may also compromise the healing of perineal wound.^[4]^

Pelvic peritoneum closure with running suture is a partition technique, which is believed to be capable of separating the abdominal and pelvic cavity, thus prevent the small bowel from descending into the pelvic dead space. Studies have demonstrated that pelvic peritoneum closure could reduce the incidence of perineal wound complications, intestinal obstruction and PH.^[5]^ However, pelvic peritoneum reconstruction (PPR) may not always be feasible, especially in patients who had received a neoadjuvant radiotherapy and suffered severe tissue fibrosis in the pelvis (a rigid pelvis) or those who have no redundant peritoneum coverage and conventional closure may under high tension.^[2,5]^

Recently we have reported a novel method, using the bladder peritoneum flap to reconstruct the pelvic peritoneum in laparoscopic ELAPE for 3 patients with a rigid pelvis after neoadjuvant radiotherapy.^[2]^ According to the IDEAL framework, which describes a pathway for testing surgical innovation, the previous technical note belongs to IDEAL stage 1 as it described proof of concept. In order to further develop the bladder peritoneum flap closure procedure, we intend to conduct an IDEAL stage 2a prospective development study.

## 2 Methods

### 2.1 Study objectives

The objective of this study is to evaluate the short-term clinical, technical and safety outcomes of PPR using the bladder peritoneum flap in laparoscopic ELAPE.

### 2.2 Study design

This is a multi-center, prospective development study. The method of PPR using the bladder peritoneum flap in laparoscopic ELAPE is at the development stage. And this protocol fulfills the requirement of IDEAL framework stage 2A.^[6]^

Approval of the ethics committee has been obtained from the ethics committee of West China Hospital, Sichuan University (2019 No. 194). The present study was registered on the clinicaltrials.gov (NCT04177407) on November 27, 2019. For adopters from other institution, approval of the local ethic committee will be required. Procedure development with reasons for changes will be presented to the institutional review board for further assessment. Benefits and risks of the study will be informed to participants. Only participants who signed an informed consent form and agree to participate will be included in this study. Participants have the right to quit the study at any time without any reason. In emergency circumstances, surgeons have the right to end the study. Data of the details will be stored in a database and published after the trial.

### 2.3 Study endpoints

The primary endpoints are the occurrence of intraoperative and postoperative complications of PPR using the bladder peritoneum flap in laparoscopic ELAPE.

Intraoperative complications include bleeding, flap devitalization, flap laceration, bladder injury and any other event that may cause the failure of the novel method. Postoperative complications include complicated perineal wound healing, PH, presacral sinus and small-bowel obstruction (SBO) within 1 year after surgery. Perineal wound healing is evaluated using the Southampton wound score system, and a score ≥ IV is regarded as complicated perineal wound healing.^[7]^ PH was defined as the presence of intra-abdominal content beyond a line connecting the coccyx and the lower margin of the pubic symphysis on sagittal views of CT.^[8]^ PH will be further classified as symptomatic and asymptomatic PH, regarding to patient's complaint of symptoms and physical signs. Presacral sinus is defined as accumulation of fluids in the presacral dead space after removal of rectum and mesorectum. SBO is classified according to the Clavien-Dindo classification^[9]^ and reasons for obstruction will be recorded (adhesive, tumor metastatic or stoma-related).

Secondary end points include:

(1) Overall complication rate within 30 days after surgery.
(2) The incidence of the presence of the small bowel in the retro-urogenital space, which was define as space between the bladder/uterus and the sacrum on axial views of CT.
(3) 1-year overall survival and tumor free survival.
(4) Quality of life at baseline, 3 months, 6 months, and 12 months after surgery.
(5) Bladder and prostatic function evaluation at baseline, 3 months, 6 months, and 12 months after surgery.

### 2.4 Population

Consecutive patients with primary or recurrent rectal cancer eligible for laparoscopic ELAPE will be included in this study. If a patient has a rigid pelvis after neoadjuvant chemoradiotherapy and the peritoneum reconstruction could not be done by a running suture, the patients will be considered as a candidate for the bladder peritoneum flap closure. A rigid pelvis is defined as a stiff pelvis with no redundant peritoneal coverage because of severe fibrosis caused by neoadjuvant radiotherapy. In addition, if PPR with a running suture is with high tension, the patient will also be included to receive the bladder peritoneum flap closure. All patients with PPR will be also included as comparison. Details of the inclusion and exclusion are formulated in the Table 1.

**Table 1:**
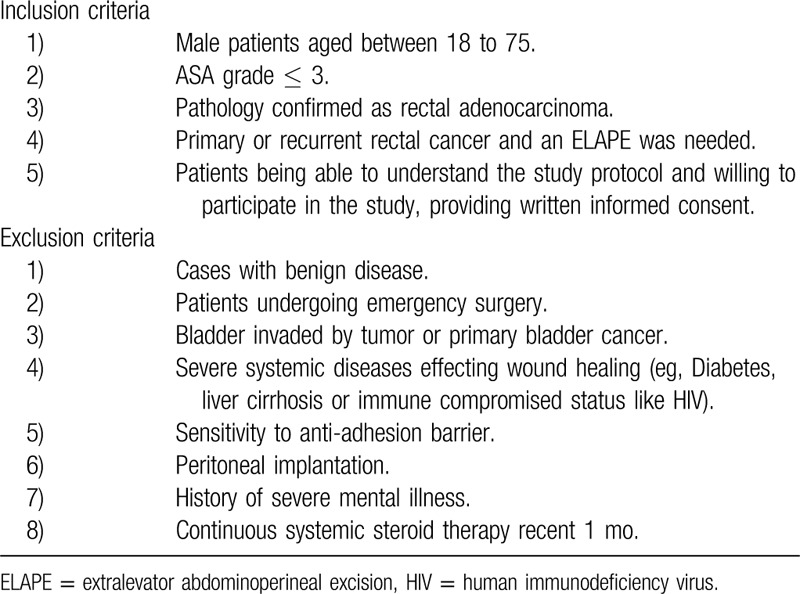
Details of the inclusion, exclusion criteria.

Usually, in a Stage 2A study, a single institution with some early adopters is involved for technique-based innovations. However, our study requires that the adopter should have sufficient experience of laparoscopic ELAPE (30 or more cases experience) and routinely perform PPR. In our center, only 1 other colorectal surgeon fit this requirement. Therefore, we will invite surgeons from other institution who meet the requirement to join us and make the present study a multicenter study.

### 2.5 Operative procedures

All the laparoscopic ELAPE should follow the principle of total mesorectal excision and the perineal part of ELAPE procedure should follow the principles proposed by Holm et al. Video of surgery should be saved for further check.

**Figure 1:**
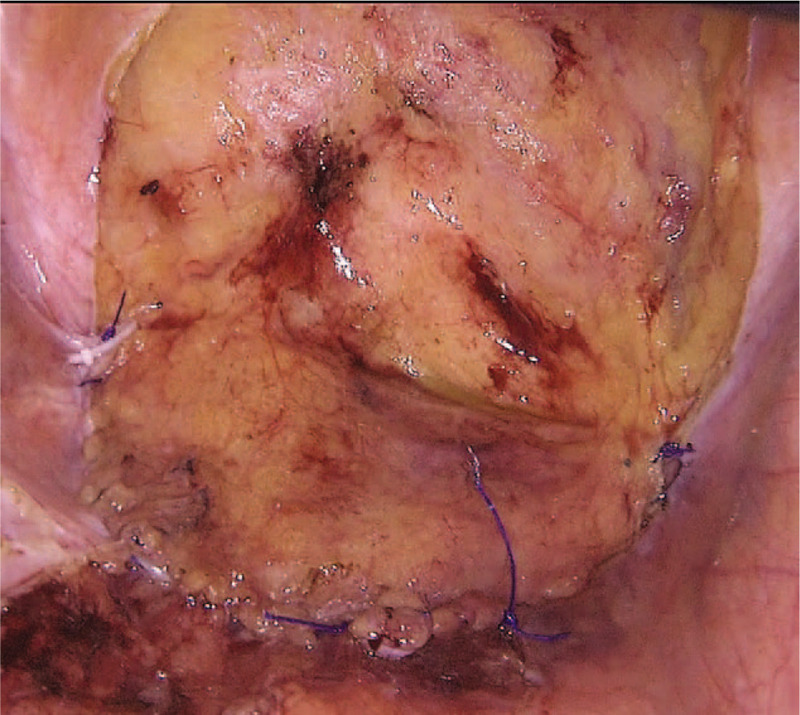
The pelvic peritoneum was reconstructed using the bladder peritoneum flap.

After transection of the rectum, if PPR with running suturing is not feasible, the bladder peritoneum flap closure is performed. The procedure of the bladder peritoneum flap closure was described in the previous stage 1 study,^[2]^ briefly, it has 3 main steps:

(1) Flap planning. In the laparoscopic view, the bladder peritoneal flap has an arch shape or “U” shape with the bottom at the anterior wall of the pelvic cavity entrance. The height of the arched flap is equal to the distance from the bladder to the sacral promontory. We measured the distance with a 15-cm suture, and then determined the highest point of the flap and marked it with electrocautery.
(2) Flap harvest. The electrocautery was set at a low power level and used in a cutting mode. The peritoneum was incised at the planned level and peeled off the bladder. Sharp dividing in close proximity to the muscular layer of the bladder, combined with blunt separation, was applied to maintain a good blood supply to the flap.
(3) Flap suturing. Rotate the harvest flap to cover the entrance of the pelvic cavity. The closure is then completed with a running suture (Prolene, Ethicon Inc, Cincinnati, OH) from the right side to the left, by suturing the free edge of the flap to the brim of the pelvis (Fig. 1).

Evolving of procedure. In the development study, 2 more steps are to add to the procedure. First, to reduce potential abdominal adhesion introduced by the rough surface of bladder and the flap, an anti-adhesion barrier (INTERCEED, Ethicon Inc, Cincinnati, OH) is then used to cover the rough surface of the bladder and the flap. Second, to determine the flap's vitality, after flap harvest, a near-infrared fluorescent dye (indocyanine green fluorescence [ICG]) is injected to the rotated flap and an imaging system is used to detect blood supply of the flap. If vascular perfusion via ICG is poor or delayed, the flap closure is considered as a failure.

For patients who are feasible for PPR with conventional running suturing, they will be still included as comparison.

### 2.6 Collection and analyses of data

Surgery related data collection. Surgical videos of all the included patients will be evaluated by the major center (Department of Gastrointestinal Surgery, West China Hospital of Sichuan University). Intraoperative information of primary tumor, operation time, data related to pelvic peritoneal reconstruction, postoperative recovery and postoperative pathology will be collected. Intraoperative complications are evaluated by operators during the surgery.

Follow-up data collection. Follow-ups will be performed by professional officers via outpatient service, phone or letter. The follow-up period is set as 1 year. Life quality is evaluated by EORTC QLQ-C30, EORTC QLQ-CR29 quality of life scale. Prostatic function is evaluated by international prostatic symptom score and bladder function is appraised by bladder residual urine. Schedule of follow-ups is listed in Table 2. Laboratory tests performed in none- research centers are accepted in the follow-up. However, radiological examination should be performed in the research centers.

**Table 2:**
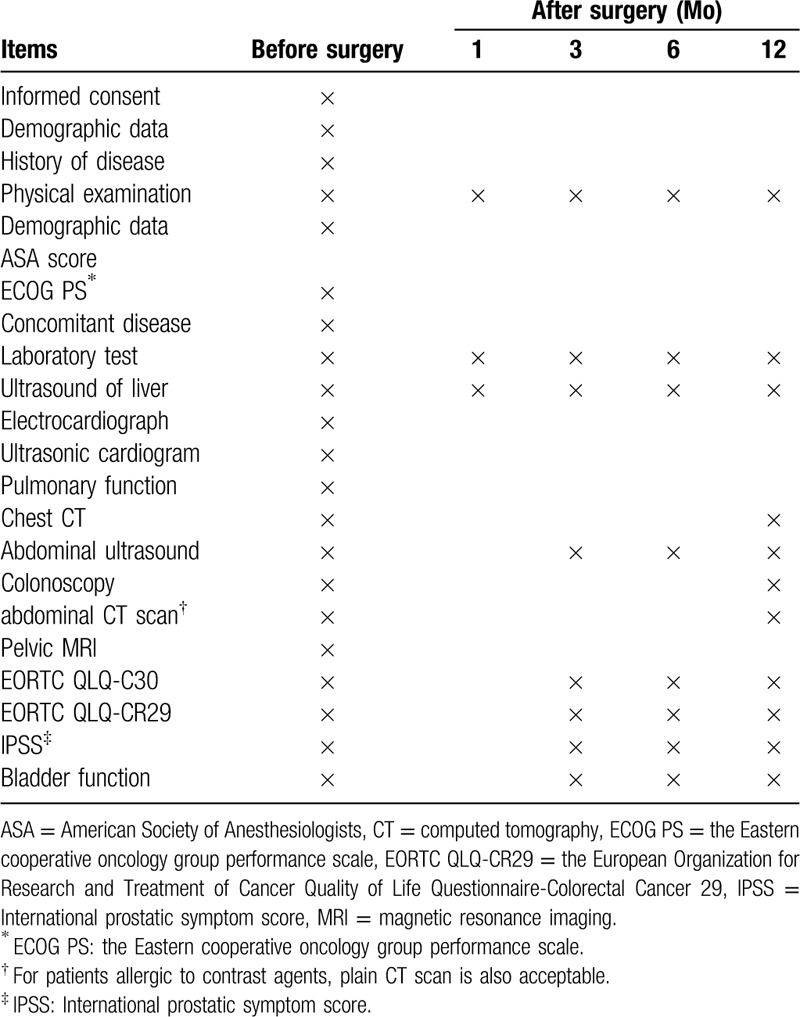
Follow up items and time.

### 2.7 Statistics

The hypothesis of our study is to test superiority of the bladder peritoneum flap closure over no closure in patients who are not eligible for PPR. The currently available literature of PPR is difficult to interpret with regard to complicated perineal wound healing, PH and SBO. Owing to lack of high-quality data in the literature, we define a clinically relevant difference in complicated perineal wound healing, PH and SBO which could justify the use of the bladder peritoneum flap closure. The difference is considered to be 30%. A total of 30 patients in the study group are needed to be able to detect a 30% reduction in the incidence of 1-year complicated perineal wound healing, PH and SBO by performing the bladder peritoneum flap closure from 45% to 15% (α = 0.05, 1-β = 0.80, superiority margin = 0.05, dropout rate = 0.1).

As stated in the part of Population, there will not be a control group. The study will be stopped if complication rate shows much higher (over 10%) than that the comparison group. Outcomes are reported in “Study endpoints” section.

### 2.8 Monitoring and reporting

Professional investigators will monitor the safety of included patients. Patients will be withdrawn from the trial if investigators believe they suffered risks based on medical reasons. Detected serious adverse events will be submitted to the related medical ethical committee for further evaluation within 1 month after the occurrence. All adverse events will be updated on the clinicaltrials.gov. Investigators can examine detail of the adverse events online.

### 2.9 Patient and public involvement

Patients or public were excluded from the design of the trial and the selection of end points parameters. Patients are excluded from the recruitment and conduct of the trial either. Results of the present study will be published via medical journal(s). Participants of the study will be informed the outcomes by local monitors via phone or letter.

## 3 Results

Patients recruitments is ongoing presently in all the centers involved in this study. Results are expected in 2021. Raw data are expected to be available to publication after the study via an open access database.

## 4 Discussion

This multi-center, prospective development study is an IDEAL framework stage 2a study. Through this stage of study, we hope to:

(1) modify the bladder peritoneum flap closure to minimize potential risks of complications;
(2) make the procedure refine enough at the end of stage 2a study to allow replication in later IDEAL stage study;
(3) evaluate if this procedure could be adopted by other surgeons successfully;
(4) evaluate the short-term clinical, technical and safety outcomes of the bladder peritoneum flap closure.

To prevent the small bowel or intraabdominal content from entering the pelvic dead space, several surgical methods have been adopted but there is no consensus on what the best method is. Generally, these methods can be divided into 2 categories: partition techniques and filling techniques. Partition techniques separate the abdominal and pelvic cavity with closed pelvic peritoneum^[5,10]^ or prosthesis.^[11]^ Filling techniques are to fill the pelvic dead space with a pedicled omentoplasty,^[12]^ myofascial flap or breast prosthesis.^[13]^ The omentoplasty seems to be the most popular technique, especially in laparoscopic ELAPE, in which suturing of pelvic peritoneum is sometimes technically demanding.^[5,10]^ However, a recent meta-analysis demonstrated that the omentoplasty is associated with increased risk for PH and perineal wound complications because non-vital omentoplasty may cause pelvic infection and subsequent complications.^[12,14]^ Traditionally, pelvic peritoneum is primarily closed after ELAPE by a running suture, barbed suture or Hem-o-lok clips.^[5,10]^ In some scenarios, reconstruction of pelvic peritoneum is difficult because of high tension. Modifications could be made to reduce the tension, such as making a shallow incision on both sides of the surface of pelvic peritoneum.^[5]^ Nevertheless, if there is no redundant pelvic peritoneum coverage, especially when the patient has a rigid pelvis after neoadjuvant radiation, either conventional or modified pelvic peritoneum closure is not feasible. Under these circumstances, even surgeons who routinely perform PPR may choose not to close the pelvic peritoneum. Currently, to our knowledge, there is no other studies on PPR regarding these situations. Our novel technique using the bladder peritoneum flap to reconstruct pelvic peritoneum in laparoscopic ELAPE could offer a new option to colorectal surgeons.

PPR is a well-accepted procedure to prevent perineal wound complications and intestinal obstruction in open abdominoperineal resection (APR).^[15]^ Despite this, there are only few evidences of application of PPR in laparoscopic APR/ELAPE. Yan et al recently reported that patients with PPR has a lower rate of perineal surgical site infection, delayed perineal healing, ileus and PH compared to patients without pelvic perineum reconstruction.^[5]^ To be noticed, in their study, the rate of PH was 0% in patients with pelvic perineum reconstruction, but it was based on data during the hospitalization. Similarly, Xu et al^[16]^ used Hem-o-lok clips to reconstruct pelvic peritoneum in laparoscopic APR for 22 patients with low rectal cancer or anorectal cancer, and there was no PH after 3 months. In the study of Wang et al,^[10]^ they compared the outcomes between patients with PPR along with primary perineal wound closure and patients with biological mesh closure for perineal wound without PPR. Their results showed that PPR was associated with shorter postoperative hospital stay and lower cost. The 1-year surgical outcomes were comparable between PPR with primary closure and biological mesh closure and there was no 1-year PH. In our previous study reporting 33 cases of laparoscopic ELAPE with PPR, there was also no symptomatic PH after 12 months follow-up and only 1 intestinal obstruction (3.0%, 1/33). Therefore, based on evidences from literatures and our own experience, we routinely perform PPR in laparoscopic ELAPE.

In the previous stage 1 study,^[2]^ we reported 3 cases using the bladder peritoneum flap to reconstruct pelvic peritoneum in patients with a rigid pelvis undergoing laparoscopic ELAPE In the past 9 months, we performed another 3 bladder peritoneum flap closure for patients with a rigid pelvis. Patient selection was extremely strict as we only perform this procedure in patients after neoadjuvant radiotherapy. As mentioned above, fibrosis in the pelvis is not the only condition for the failure of conventional PPR. In obesity patients or patients with bulky tumor, the pelvic perineum coverage may also be insufficient.^[5]^ Therefore, to accelerate the inclusion of patients, we would expand our inclusion criteria and not limit to patients with neoadjuvant radiation.

In this prospective development study, we would make some modifications, with the intention to minimize the potential risks of the bladder peritoneum flap closure. First, we would use an anti-adhesion barrier to protect the rough surface of the bladder and the flap. A study of Watanabe^[17]^ showed that the usage of anti-adhesion can reduce the incidence of postoperative adhesive SBO (0.0% vs 4.6%). Guidelines also suggest the usage of adhesion barriers as adjuvants in peritoneal reconstruction.^[18]^ We hope the application of anti-adhesion barrier could lower the risk for adhesive SBO. Second, we will use intraoperative ICG fluorescence after the bladder peritoneum flap closure to evaluate the vitality of the flap, if the vascular perfusion via ICG is poor or delayed, which indicates devitalization of the flap, the case will be counted as a failure and the pelvic peritoneum will not be closed thereafter. The incidence of flap devitalization would be objective parameter to reflect if the adopters have mastered the flap harvest step. Last, during the stage 2 study, further evolving of procedure may be utilized and will be reported in detail in the final article.

In conclusion, the bladder peritoneum flap closure may serve as an option to reconstruct pelvic peritoneum in patients who are not eligible for conventional pelvic peritoneum closure in laparoscopic ELAPE, but proof of which is much needed.

## Author contributions

Yu Shen and Tinghan Yang drafted the manuscript. Xiangbing Deng and Jinliang Yang edited the first manuscript. Wenjian Meng and Ziqiang Wang supervised this protocol. All authors read and approved the final manuscript.
